# Optical imaging of pre-invasive breast cancer with a combination of VHHs targeting CAIX and HER2 increases contrast and facilitates tumour characterization

**DOI:** 10.1186/s13550-016-0166-y

**Published:** 2016-02-10

**Authors:** Marta M. Kijanka, Aram S. A. van Brussel, Elsken van der Wall, Willem P. T. M. Mali, Paul J. van Diest, Paul M. P. van Bergen en Henegouwen, Sabrina Oliveira

**Affiliations:** Division of Cell Biology, Department of Biology, Science Faculty, Utrecht University, Utrecht, The Netherlands; Department of Pathology, University Medical Center Utrecht, Utrecht, The Netherlands; Division of Internal Medicine and Dermatology, University Medical Center Utrecht, Utrecht, The Netherlands; Department of Radiology, University Medical Center Utrecht, Utrecht, The Netherlands

**Keywords:** CAIX, HER2, Nanobody or VHH, Optical molecular imaging, Fluorescence pathology, Breast cancer

## Abstract

**Background:**

Optical molecular imaging is an emerging novel technology with applications in the diagnosis of cancer and assistance in image-guided surgery. A high tumour-to-background (T/B) ratio is crucial for successful imaging, which strongly depends on tumour-specific probes that rapidly accumulate in the tumour, while non-bound probes are rapidly cleared. Here, using pre-invasive breast cancer as a model, we investigate whether the use of combinations of probes with different target specificities results in higher T/B ratios and whether dual-spectral imaging leads to improvements in tumour characterization.

**Methods:**

We performed optical molecular imaging of an orthotopic breast cancer model mimicking ductal carcinoma in situ (DCIS). A combination of carbonic anhydrase IX (CAIX)- and human epidermal growth factor receptor 2 (HER2)-specific variable domains of the heavy chain from heavy-chain antibodies (VHHs) was conjugated either to the same fluorophore (IRDye800CW) to evaluate T/B ratios or to different fluorophores (IRDye800CW, IRDye680RD or IRDye700DX) to analyse the expression of CAIX and HER2 simultaneously through dual-fluorescence detection. These experiments were performed non-invasively in vivo, in a mimicked intra-operative setting, and ex vivo on tumour sections.

**Results:**

Application of the CAIX- and HER2-specific VHH combination resulted in an increase of the T/B ratio, as compared to T/B ratios obtained from each of these single VHHs together with an irrelevant VHH. This dual tumour marker-specific VHH combination also enabled the detection of small metastases in the lung. Furthermore, dual-spectral imaging enabled the assessment of the expression status of both CAIX and HER2 in a mimicked intra-operative setting, as well as on tumour sections, which was confirmed by immunohistochemistry.

**Conclusions:**

These results establish the feasibility of the use of VHH ‘cocktails’ to increase T/B ratios and improve early detection of heterogeneous tumours and the use of multispectral molecular imaging to facilitate the assessment of the target expression status of tumours and metastases, both invasive or non-invasively.

**Electronic supplementary material:**

The online version of this article (doi:10.1186/s13550-016-0166-y) contains supplementary material, which is available to authorized users.

## Background

Molecular imaging of cell surface markers has become an increasingly important strategy for the imaging of cancer, which may be used for diagnosis, for assessment of therapy response and for guidance during surgical resection [1]. Optical molecular imaging has recently attracted much attention, because the employed probes are non-radioactive, and recent camera systems enable high resolution imaging. One of the concerns raised by optical molecular imaging is the limited sensitivity, due to restricted light penetration into the tissue, which prevents applications as whole-body imaging. Optical imaging is, however, perfectly suited for non-invasive detection of superficial tumours (e.g. breast or head and neck cancers) or tumours accessible using an endoscope (e.g. lung cancer, tumours located in the gastrointestinal tract or abdominal cavity). At the moment, under clinical evaluation in several centres, the implementation of optical molecular imaging into clinical practice could complement current protocols and facilitate non-invasive detection of cancer and monitorization of therapy response (ClinicalTrials.gov; NCT02113202, NCT01972373, NCT01508572, NCT02129933, NCT01987375). In addition, it could allow accurate delineation of tumours during surgery, for radical resections of tumours and their neighbouring precursors with minimal removal of the surrounding normal tissue. Moreover, optical molecular imaging could likely advance current pathology through ex vivo analysis of biopsies.

The basis of optical imaging lies in the detection of light emitted from fluorophore, making it a cost-effective, non-radioactive imaging modality for the detection of cancer, both in the screening and intra-operative setting. Recent advances in optical imaging probes are focused on the development of near-infrared (NIR) fluorophores. Fluorophores emitting light in the NIR range of the spectrum (e.g. 700 and 800 nm) allow deeper tissue penetration than the fluorophores which emit in the standard 400–600-nm range. These advantages are a result of lower light absorption by blood and other tissue components, as well as of minimal tissue autofluorescence in this range of the spectrum [2–4].

Besides an appropriate NIR fluorophore and an imaging system able to detect the light emitted by this fluorophore, optical molecular imaging requires high tumour specificity, which can be obtained by employing fluorescent probes targeting tumour-specific markers that are ideally (over)expressed strictly in cancerous, and not in normal, tissues. Several biomarkers have been identified and their (over)expression has been associated with cancer development. Among these are the human epidermal growth factor receptor 2 (HER2) and the hypoxia marker carbonic anhydrase IX (CAIX) [5, 6]. Various targeting moieties have been employed to facilitate tumour targeting, such as affibodies, peptides, conventional monoclonal antibodies (mAbs) or antibody fragments. Previously, we reported successful optical molecular imaging using the variable domain of the heavy chain from heavy-chain antibodies (VHH, also referred to as nanobodies) conjugated to the NIR fluorophore IRDye800CW in xenograft mouse models [7, 8]. VHHs, which are the smallest, naturally occurring, functional antigen binding fragments of only 15 kDa, proved to be very promising tumour targeting agents. Being a tenth of the molecular weight of mAbs and having high binding affinity and specificity, VHHs efficiently penetrate throughout the tumour mass and are retained at the tumour. Due to the rapid accumulation of these nanobodies into the tumour and their rapid clearance (half-life of 1–2 h [8, 9]), its visualization is already possible 2–4 h postinjection (p.i.). In contrast, mAbs may require more than 24–48 h to accumulate at the tumour and provide comparable contrast.

Sufficient signal (described as contrast or tumour-to-background ratio, i.e. T/B ratio) and a clear delineation of the tumour are essential for successful and accurate tumour detection through imaging. Taking into account the heterogeneity of cancers (e.g. breast cancers [10]), it may be a challenge for a unique probe to provide enough signal for optical imaging of early-stage cancers. We hypothesized that the combination of two tumour-specific probes could increase the T/B ratio and thus facilitate tumour detection. Moreover, by using dual-spectral imaging, information may be obtained about expression levels of different tumour markers within the same tumour, which could accelerate tumour characterization.

In this study, we investigate whether a combination of two optical probes that specifically recognize two independent breast cancer markers could improve tumour detection by optical imaging. For this, we have employed the VHH B9 targeting CAIX, which localizes to peri-necrotic regions of tumours [11], and the VHH 11A4 targeting HER2, which is known to have a more homogenous distribution throughout the tumour tissue [7]. These VHHs were selected based on their specificity and binding affinity for their targets. B9 binds to CAIX with an affinity of approximately 7 nM and it did not bind to cells devoid of expression of CAIX nor to cells expressing CAIX when in the presence of high concentrations of the CAIX ectodomain [11]. 11A4 binds to HER2 with an affinity of approximately 500 pM, and its specificity was demonstrated by the absence of binding to the ectodomains of HER1, HER3 and HER4 and also the absence of binding to cells that did not express HER2 [7]. Subsequently, preclinical studies have shown the rapid accumulation of these VHH probes in tumours and have confirmed their specificity: 11A4 accumulated in the SkBr3 HER2-positive subcutaneous tumour model, whereas no accumulation was observed in the MDA-MB-231 HER2-negative model [7]. B9 accumulated in a ductal carcinoma in situ (DCIS) orthotopic model, to an extent that correlated with the expression level of CAIX [11]. In the present study, both VHHs were conjugated to the same fluorophore, i.e. IRDye800CW, and the T/B ratios after co-injection of both VHHs were compared in a breast cancer tumour model with the T/B ratios obtained after co-injection of either of the VHHs together with an irrelevant VHH, R2. In addition, B9 and 11A4 were conjugated to different NIR fluorophores to investigate whether this approach could be used to detect the expression status of CAIX and HER2 in vivo. This combination of VHHs was also evaluated ex vivo in a mimicked surgical context and for the detection of lung metastases through dual-spectral optical imaging. Finally, immunohistochemistry was performed to validate fluorescent 11A4 and B9 probe localization through dual-spectral optical imaging of tumour sections. Our data have revealed improved imaging by using a combination of different nanobody-based optical probes.

## Methods

### Cell lines and cell culture conditions

MCF10DCIS.com (further referred to as MCF10DCIS) cells (Asterand, Detroit, MI, USA), mimicking DCIS of breast, were cultured according to the supplier’s guidelines in Dulbecco’s modified Eagle medium nutrient mixture F-12 (DMEM/F12) supplemented with 10 % (*v*/*v*) fetal calf serum (FCS), 100 IU/ml penicillin, 100 μg/ml streptomycin and 2 mM l-glutamine at 37 °C in a humidified atmosphere containing 5 % CO_2_. The generation of luciferase overexpressing MCF10DCIS cells has been described before [12].

### VHH production and purification

The HER2-specific VHH, 11A4, and the CAIX-specific VHH, B9, were produced as described before [7, 11]. Briefly, VHH genes were re-cloned into the pQVQ72 expression vector (kindly provided by QVQ BV), which enables site-directed conjugation of IRDye680RD maleimide and IRDye800CW maleimide (LI-COR Biosciences, Lincoln, Nebraska, USA). Irrelevant VHH, R2, was produced as described previously [8]. Bacterial production of the VHHs was induced by addition of 1 mM isopropyl-beta-d-thiogalactopyranoside (IPTG) when bacteria reached log phase. HER2- and CAIX-specific VHHs were purified from the periplasmic fraction by protein A affinity chromatography using a HiTrap protein A HP column (GE Healthcare, Zeist, The Netherlands) on the ÄKTAxpress system (GE Healthcare, Zeist, The Netherlands). Irrelevant VHH (R2) was purified using immobilized metal affinity chromatography (IMAC; Talon; Takara Bio Europe/Clontech, Saint-Germain-en-Laye, France).

### Conjugation of the NIR fluorophores

Conjugation of the fluorophores was performed as described before [7]. Briefly, 11A4 and B9 VHHs were treated with 20 mM Tris(2-carboxyethyl)phosphine (TCEP) in 50 mM Tris-HCl pH 8.5 for 15 min at room temperature (RT), then the buffer was replaced to 0.4 mM ethylenediaminetetraacetic acid (EDTA) in phosphate-buffered saline (PBS, Lonza, Basel, Switzerland) using a size-exclusion chromatography resin in Pierce Zeba™ Desalting Spin Columns (Thermo Fisher Scientific, Landsmeer, The Netherlands) and incubated with a threefold molar excess of fluorophore at 4 °C for 16 h. 11A4 was either conjugated to IRDye680RD maleimide (further referred to as 11A4-680) or to IRDye800CW maleimide (further referred to as 11A4-800), while B9 was conjugated exclusively to IRDye800CW maleimide (B9-800). The irrelevant VHH, R2, was randomly conjugated to *N*-hydroxysuccinimide (NHS) esters of either IRDye800CW or IRDye700DX (referred to as R2-800 and R2-700, respectively) in twofold molar excess of NIR fluorophore to protein, for 2 h at RT. All fluorophores were purchased at LI-COR Biosciences (Lincoln, Nebraska, USA). After conjugation, the remaining unconjugated fluorophores were removed using sequentially two (in case of IRDye800CW) or three (in case of IRDye680RD or IRDye700DX) Pierce Zeba™ Desalting Spin Columns (Thermo Fisher Scientific, Landsmeer, The Netherlands). The degree of conjugation was determined according to the instructions given by the provider for each of the fluorophores.

### In vivo studies

Ethics statements: All procedures performed in studies involving animals were in accordance with the ethical standards and approved by the Animal Experimental Committee of Utrecht University (DEC-Utrecht no. 2012.III.02.015).

The orthotopic mouse model of breast cancer (MCF10DCIS cells) used in this study was based on a previously described model [13]. In brief, SCID/Beige female mice (6 weeks old) were purchased from Harlan. Animals were housed in standard Perspex cages with free access to food and water. To reduce food-induced fluorescence in the intestinal tract, animals received a chlorophyll-free diet 2 weeks prior to imaging (Harlan Laboratories, The Netherlands). In order to eliminate autofluorescence by the fur, mice were shaved in the abdominal and upper leg area and treated with Veet cream (local pharmacy) to remove remaining hair. Forty thousand MCF10DCIS cells were inoculated in the fourth mammary glands at both sides of mice. After tumour formation (approximately 8–9 weeks after inoculation of cells), mice carrying xenografts with diameters of approximately 0.5 cm were subjected to imaging.

In the first part of the study, where the advantage of using two probes instead of one was tested with respect to obtaining higher tumour-to-background ratios (T/B ratios), mice were divided into three groups. Each group was injected intravenously with one of the following combinations of VHHs (50 μg of each VHH-IR, thus 100 μg in total): 11A4-800 and B9-800 (*n* = 7), 11A4-800 and R2-800 (*n* = 7) or B9-800 and R2-800 (*n* = 7). After the injections, mice were imaged with the Pearl Impulse Small Animal Imaging System (LI-COR Biosciences) at the following time points: 30 min, 1 h, 2 h, 3 h, 4 h, 5 h, 6 h, 24 h and 48 h postinjection (p.i.). Regions of interest (ROIs) were drawn in the tumour and in the upper leg region as normal tissue areas. The upper leg region was chosen as the background due to the proximity to the abdominal area and the reduced risk for the mice, as compared to the risk of shaving the thoracic breasts. T/B ratios were calculated by dividing the mean intensity of tumour ROI by the mean intensity of background ROI (drawn in normal tissue area) determined with Pearl Impulse Software (v.2.0, LI-COR Biosciences).

In the second part of the study, mice were divided into three groups and then injected intravenously with combinations of VHHs conjugated to different NIR fluorophores to study whether the expression status of HER2 and CAIX could be detected simultaneously in vivo (50 μg of each VHH-IR, thus 100 μg in total): 11A4-680 and B9-800 (*n* = 8), 11A4-800 and R2-700 (*n* = 8) or B9-800 and R2-700 (*n* = 7). After the injections, mice were imaged as described above and the fluorescence intensities of IRDye680RD and IRDye700DX were detected in the 700-nm channel, whereas the fluorescence intensity of IRDye800CW was detected in the 800-nm channel. T/B ratios were calculated as described above.

In the third part of the study, the potential of VHH-IR to detect lung metastasis was evaluated. For the development of the lung metastasis model, 1 × 10^5^ MCF10DCIS cells were injected intravenously through the tail vein. Having confirmed the presence of lung metastasis in a non-invasive manner by bioluminescence imaging (Photon Imager, Biospace Labs, Paris, France), mice were injected intravenously with the combination (50 μg of each VHH-IR, 100 μg in total) of 11A4-680 and B9-800 (*n* = 3). As controls, one mouse was injected with R2-800 and R2-700 and a healthy mouse with 11A4-680 and B9-800. Mice were sacrificed 5 h p.i. by cervical dislocation, and lung metastases were imaged with the Pearl Impulse Small Animal Imaging System (LI-COR Biosciences) upon surgical removal of lungs.

### Imaging of fluorescent sections and immunohistochemistry

Mice injected with the combination of VHHs with either IRDye800CW, IRDye680RD or IRDye700DX were sacrificed 5 h p.i., and their tumours were collected. These were first imaged (i.e. ex vivo) and, immediately thereafter, fixed in neutral buffered formalin, routinely processed to paraffin blocks and stored in the dark until further processing. Four-micrometre-thick sections were scanned using the Odyssey imaging system at the highest (21 μm) resolution and quality. Immunohistochemistry (IHC) for detection of CAIX and HER2 combined with a haematoxylin and eosin (H&E) staining were performed as described before [10]. Slides were scanned with the Scanscope XT 120 scanner (Aperio, Vista, CA, USA).

### Statistics

Statistical analysis was performed using GraphPad Prism (version 5.02). Comparisons of T/B ratios were analysed using a Mann-Whitney test. *p* values of <0.05 were considered to be statistically significant.

## Results

### Preparation of HER2- and CAIX-specific VHHs conjugated to NIR fluorophores

In order to investigate whether a combination of two fluorescent VHHs specifically recognizing two separate and validated breast cancer biomarkers could (a) improve tumour detection through optical imaging with an increase in T/B ratio and/or (b) facilitate tumour characterization and observation of different areas of the tumour, we used the HER2-specific VHH, 11A4 [7], and the CAIX-specific VHH, B9 [11]. Similarly to our previous studies [7, 11], in order to prevent any effect of the fluorophore conjugation on the binding affinity, both VHHs were site-specifically conjugated to their respective fluorophores using a cysteine that was introduced in the C-terminal region. Besides this extra cysteine, most VHHs possess two cysteines that form a disulfide bridge, which is inside the structure of the folded protein, and contributes to protein integrity. In this study, 11A4 was site-specifically conjugated to either maleimide IRDye800CW or IRDye680RD (named 11A4-800 and 11A4-680, respectively), whereas B9 was conjugated to IRDye800CW only (named B9-800). In all in vivo experiments, we have included an irrelevant VHH, R2, as a control. As the binding affinity of this control VHH is of less importance, R2 was randomly conjugated to either IRDye800CW or IRDye700DX (referred to as R2-800 and R2-700, respectively). After purification of the fluorescent conjugates, the amount of free fluorophore remaining in the sample was less than 5 % of the total fluorophore and thus these conjugates were considered suitable for use in the in vivo study (determined by sodium dodecyl sulfate polyacrylamide gel electrophoresis (SDS-PAGE), Additional file 1: Figure S1). The degree of conjugation of each of the fluorophores to each VHH was 0.5, which is in agreement with previously reported data [7, 8].

### In vivo single-spectrum imaging using a combination of VHHs

**Fig. 1:**
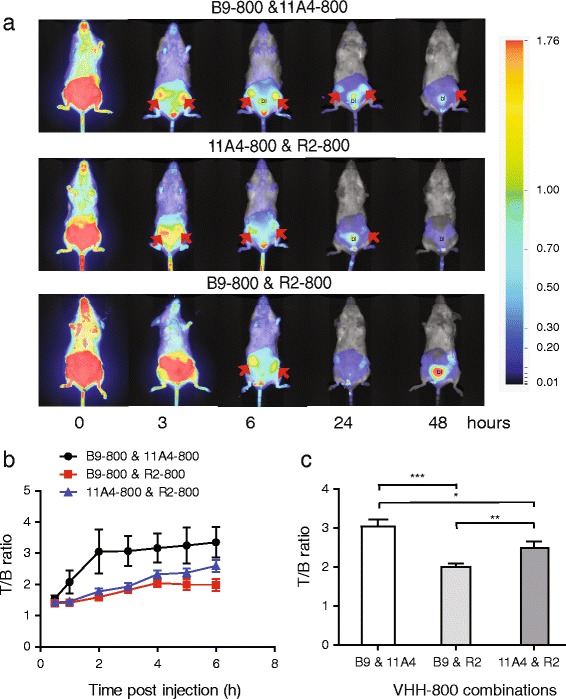
Use of a combination of two tumour-specific VHHs for optical imaging results in higher T/B ratios. **a** Optical imaging of mice xenografted with MCF10DCIS cells in time upon injection of the combination 11A4-800 and B9-800 (*upper panel*) and the control combinations, 11A4-800 and R2-800 (*middle panel*) or B9-800 and R2-800 (*bottom panel*). *Arrows* point to tumours, *bl* depicts bladder. **b** T/B ratios in time (*n* = 7 per group). Graph represents mean ± SEM. These were calculated from ROIs drawn around each tumour and non-tumour area. **c** Comparison of overall T/B ratios and their SEM obtained at all time points for animals injected with B9-800 and 11A4-800 and the controls: B9-800 and R2-800 or 11A4-800 and R2-800. The *p* values were determined, and statistical significance is indicated by * when *p* < 0.05, ** when *p* < 0.01 and *** when *p* < 0.0001

To investigate whether the targeting of the tumour with two tumour-specific VHHs conjugated to the same fluorophore results in an increased contrast, we injected intravenously seven mice bearing two MCF10DCIS breast cancer xenografts in the fourth mammary glands with either 11A4-800 and B9-800 or the controls 11A4-800 and R2-800 or B9-800 and R2-800. The imaging was performed up to 48 h postinjection (p.i.). Already 3 h p.i., a clear accumulation of the fluorescent probes was seen in the tumours for each of the three probe combinations (Fig. 1a, red arrows). NIR fluorescence was also found at the bladder, which is expected due to the rapid renal clearance of the VHH. Accumulation of the NIR fluorescence at the kidneys was not visible, because mice were imaged ventrally. Fluorescence intensities were determined for each individual tumour (two tumours per mouse of seven mice in total) and a non-tumour tissue in the leg area, in such a way that the signal was not affected by the signal of the tumour or bladder (i.e. background). The T/B ratios were calculated and plotted in time (Fig. 1b). Over time, the background fluorescence decreased as a result of rapid clearance of the probes, which led to an increase in contrast expressed as T/B ratios. After combining T/B ratios obtained at all time points, an overall significant difference in T/B ratio increase was found between the group injected with 11A4-800 and B9-800 and the control groups injected with either 11A4-800 and R2-800 (22 % increase in T/B ratio) or the B9-800 and R2-800 cocktail (51 % increase in T/B ratio) (Fig. 1b). Interestingly, we also observed a significant difference between the two control groups, which indicated that the signal obtained with the HER2-specific VHH was higher than that obtained with the CAIX-specific VHH.

### In vivo dual-spectral imaging using a combination of VHHs

**Fig. 2:**
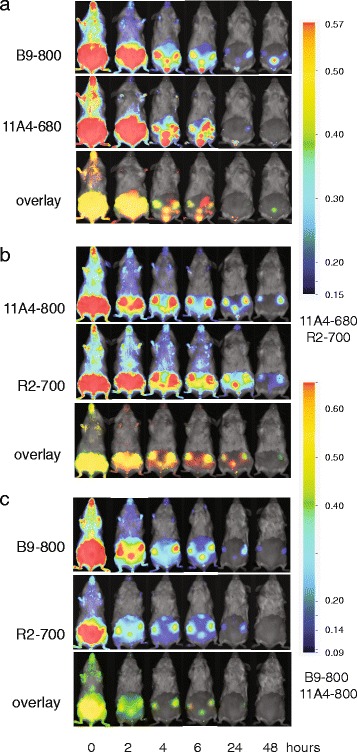
Dual spectral molecular imaging using tumour-specific VHHs allows for simultaneous expression status determination of two different tumour markers, namely CAIX (targeted by VHH B9) and HER2 (targeted by VHH 11A4). Irrelevant VHH R2-700 was used as a negative control. Mice xenografted with MCF10DCIS cells were imaged in time upon injection of the following: **a** tumour-specific combination consisting of 11A4-680 and B9-800, **b** control combination consisting of 11A4-800 and R2-700 and **c** control combination consisting of B9-800 and R2-700. The observed *yellow colour* present in the overlays in each of the *bottom panels* originates from the overlay of *green* and *red signals*

To determine whether two independent VHHs conjugated to two different NIR fluorophores have the potential to determine the expression levels of two relevant breast cancer markers in vivo, mice were injected intravenously with B9-800 and 11A4-680 or with the controls B9-800 and R2-700 and 11A4-680 and R2-700. As expected, tumour xenografts were visible already 2 h p.i.; however, the background fluorescence was also high at that time point (Fig. 2a–c) probably due to incomplete clearance of non-bound fluorescent VHHs. Clear delineation of the tumours was possible 4 h p.i. The irrelevant VHH, R2-700, was also found at the tumour site, but this occurred to a lesser extent as compared to the tumour-specific fluorescent VHHs (Fig. 2b, c). Both 11A4-800 and B9-800 specifically accumulated at the tumour when co-injected with irrelevant VHH, in a similar manner to the co-injection of these tumour-specific VHHs (Fig. 2).

**Fig. 3:**
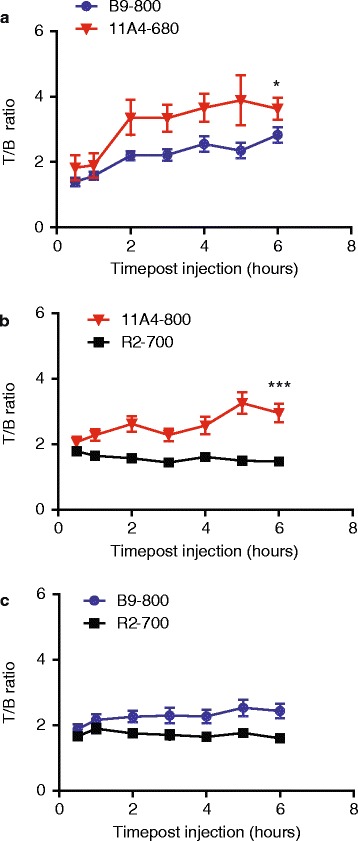
Rapid increase of T/B ratio in the first 6 h p.i. allows fast imaging upon injection of the following: **a** B9-800 and 11A4-680 (*n* = 8), **b** 11A4-800 and R2-700 (*n* = 8) and **c** B9-800 and R2-700 (*n* = 7). Graphs represent mean ± SEM. At 6 h p.i, the *p* values were determined and statistical significance is indicated by * when *p* < 0.05 and *** when *p* < 0.0001

The T/B ratios were calculated and plotted in time, revealing that the maximal T/B ratios were obtained already 2 h p.i. (Fig. 3). The T/B ratios of the irrelevant VHH, R2-700, were close to 1 (the average T/B ratio was equal to 1.56 ± 0.41) and showed no increase in time, suggesting no specific accumulation of the irrelevant VHH at the tumour. To enable sufficient removal of non-bound fluorescent VHH and therefore decrease of background intensity levels, 6 h p.i. was selected as an optimal imaging time point. A significant difference between the 11A4-680 and B9-800 T/B ratios was obtained 6 h p.i. (Fig. 3a; *p* = 0.0438). Significant differences were also observed between 11A4-800 and the irrelevant VHH, R2-700 (Fig. 3b; *p* = 0.0001). The difference between B9-800 and the irrelevant VHH, R2-700, at 6 h p.i. was of borderline significance (Fig. 3c; *p* = 0.0582).

### Dual-spectral imaging in the mimicked surgical setting

**Fig. 4:**
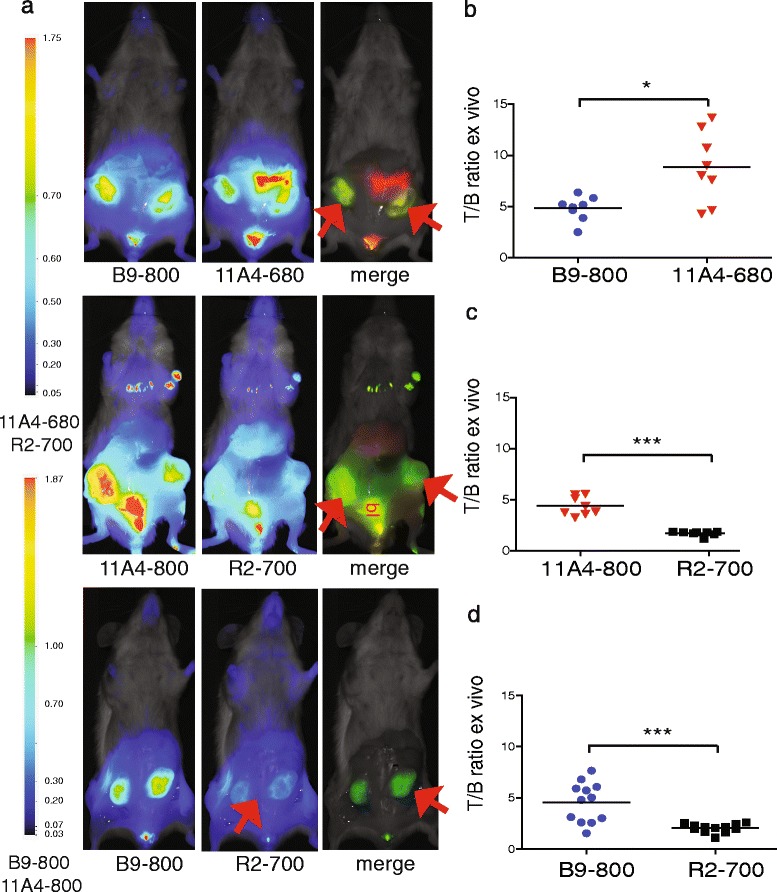
Dual-spectral imaging with two tumour-specific VHHs allows simultaneous tumour molecular status determination in an invasive, mimicked intra-operative setting. **a** Five hours p.i. of 11A4-680 and B9-800 (*left*), 11A4-800 and R2-700 (*middle*) or B9-800 and R2-700 (*right*) mice were sacrificed by cervical dislocation and their skin removed to mimic image-guided surgery setting. **b–d** T/B ratios were calculated for tumours imaged in the mimicked intra-operative setting. *Bars* represent mean, *n* = 4 mice in groups 11A4-800 and R2-700 and B9-800 and 11A4-680, *n* = 6 mice in group B9-800 and R2-700. Statistical significance is indicated by * when *p* < 0.05 and *** when *p* < 0.0001

To investigate the possible application of dual-spectral imaging in the surgical setting, a number of mice bearing MCF10DCIS xenografts were injected with the indicated fluorescent VHH combinations, and 5 h p.i. mice were sacrificed, the skin was removed thereby uncovering the xenografts and optical imaging was performed (Fig. 4a). The following VHH combinations were injected: B9-800 and 11A4-680, B9-800 and R2-700, and 11A4-800 and R2-700. As expected, a clear accumulation of IR fluorescence was found at the tumour site, for the HER2- and CAIX-specific probes. T/B ratios of 11A4-680 were approximately 73 % higher than T/B ratios determined upon injection of B9-800 (Fig. 4b). The T/B ratios of tumour marker-specific VHHs, i.e. 11A4-800 and B9-800, were approximately twofolds higher than those of the irrelevant VHH.

Compared to the results obtained with in vivo imaging (Fig. 3), the T/B ratios obtained after imaging tumours in this mimicked invasive manner were approximately twofolds higher at each time point p.i. for the 11A4 and B9 VHHs, whereas no differences in T/B ratios were observed in the case of the control R2-700 analysed in these two manners.

### Dual-spectral imaging of lung metastases ex vivo

**Fig. 5:**
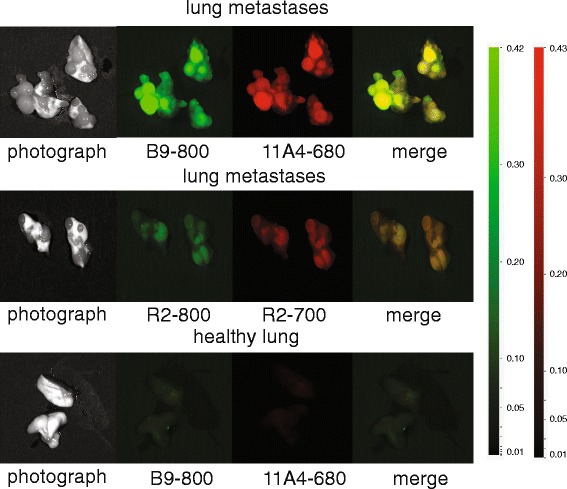
Optical molecular imaging with two tumour-specific VHHs allows detection of lung metastasis ex vivo. Optical imaging of metastasized lungs 5 h p.i. of B9-800 and 11A4-680 combination (*upper panel*) or the control R2-800 and R2-700 (*middle panel*). Optical imaging of healthy lungs 5 h p.i. of B9-800 and 11A4-680 (*lower panel*). *Yellow colour* in the overlay panel corresponds to the overlay of *green* (B9-800) and *red* (11A4-680) *colours*

MCF10DCIS cells are known to have the capacity to metastasize into the lungs. In this part of the study, the VHH combination B9-800 and 11A4-680 was evaluated for its potential to detect metastasis in the surgical setting. This could provide valuable information on their tumour marker expression status and its molecular resemblance to the primary tumour. When metastases of 1–2 mm were detected through BLI, mice were injected with the B9-800 and 11A4-680 combination. As controls, one mouse with developed lung metastases was injected with R2-800 and R2-700 irrelevant VHH combination and a healthy mouse without metastases was injected with B9-800 and 11A4-680 combination. Five hours p.i., all mice were sacrificed and their chest wall was removed in order to facilitate the imaging of the lungs. No NIR fluorescence was found in the healthy lung controls, thus confirming the tumour marker specificity of both B9-800 and 11A4-680. A clear fluorescent signal was observed at the lung bearing metastasis when the mouse was injected with the B9-800 and 11A4-680 VHH combination, which was twice the intensity of the R2-800 and R2-700 irrelevant VHH combination (Fig. 5).

### Dual-spectral imaging of tumour sections and immunohistochemical validation

**Fig. 6:**
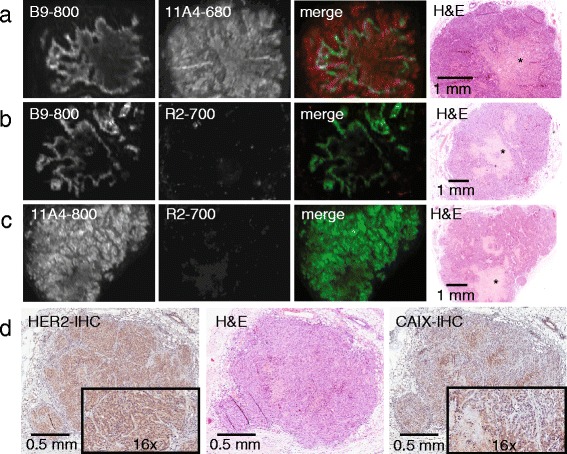
Dual-spectral fluorescence molecular pathology allows simultaneous determination of HER2 and CAIX expression status. Detection of fluorescence on tumour sections obtained from mice 5 h p.i. of the combinations **a** 11A4-680 and B9-800, **b** 11A4-800 and R2-700 and **c** B9-800 and R2-700. The fluorescence detected with the odyssey scanner is depicted in *green* for channel 800 nm (IRDye800CW) and in *red* for channel 700 nm (IRDye680RD and IRDye700DX). H&E staining of the corresponding section is depicted, where an *asterisk* indicates necrotic area. **d** IHC on sections of tumour xenografts depicting expression of HER2 and CAIX

To investigate the possibility of the ex vivo assessment of the molecular status of the tumour by employing the 11A4-680 and B9-800 combination, dual-spectral imaging of tumour sections was conducted and compared to conventional haematoxylin and eosin (H&E) staining. The HER2-targeted probe, 11A4-680, was observed to be homogenously distributed throughout the tumour section, while the CAIX-targeted one, B9-800, was only confined to the peri-necrotic areas of the tumour (Fig. 6a, asterisks (*) showing necrotic areas). Tumour sections obtained from mice that were injected with control combinations, B9-800 and R2-700, and 11A4-800 and R2-700, only presented NIR fluorescence signal in the 800-nm channel. The distribution pattern of B9-800 and 11A4-800 from these control combinations was similar to the one observed for the 11A4-680 and B9-800 combination (Fig. 6b, c, respectively). These results confirm the specific uptake of both HER2- and CAIX-targeted VHHs. In parallel, IHC analysis of the tumour sections confirmed HER2 expression of the tumours by a 2+ HER2 DAKO score for expression which was homogenously distributed throughout the tumour section, while CAIX expression was confined to the peri-necrotic tumour areas (Fig. 6d). These results validate the specificity of these VHHs for dual-spectrum imaging on tumour tissue sections.

## Discussion

Despite many efforts made over the years to facilitate cancer detection, improvements are still eagerly awaited. At the moment, six imaging modalities can be used to detect and stage cancer, namely the following: x-ray (computed tomography, CT), magnetic resonance imaging (MRI), single-photon emission tomography (SPECT), positron emission tomography (PET), ultrasound (US) and, more recently, clinical evaluation initiated with optical imaging. Each of these techniques has advantages but also limitations. Recent advances in targeted probe development stimulated further improvements of PET, SPECT and more recently also of optical imaging [14, 15]. Although the advantages of optical imaging are clear (e.g. no ionizing radiation thus no need of protection for personnel; no radioactive decay of probe thus longer stability), the sensitivity of this technique is still a concern, especially as a result of the limited penetration of light into tissues, which restricts its application to non-invasive detection of superficial tumours (or tumours accessible using an endoscope), image-guided surgery or fluorescence imaging of tissue sections. We have previously demonstrated that VHHs provide better T/B ratios than conventional antibodies at earlier time points p.i. [7, 8]. As a result of their small size, VHHs accumulate more rapidly in the tumour, and the non-bound fraction is more rapidly cleared. In fact, very recently, the first report was published on a phase I clinical trial of a VHH targeting HER2 for assessment of HER2 expression in breast cancers through PET [16]. The encouraging results obtained warrant further evaluation in a phase II trial and highlight the potential of the VHHs as probes, also for whole-body imaging when combined with different imaging modalities.

Taking into account the heterogeneity of (pre-invasive) breast cancers [10] as a model, here, we investigated whether the combination of two fluorescent VHHs targeting two independent and validated tumour markers could improve tumour detection through optical imaging by an increase in T/B ratios. The employed VHH combination consisted of a VHH binding to HER2 and a VHH binding to CAIX. The performance of this combination was evaluated in an orthotopic tumour model mimicking the comedo subtype of DCIS, which, as we described earlier [12], is associated with a relatively high risk of local recurrence and progression to invasive cancer. Crucial for these studies is the similar conjugation efficiency of the fluorophore to the two VHHs. This could allow comparison between the different groups based on the fluorescence intensity measured. To avoid any detrimental effects of fluorophore conjugation on the binding capacity of the VHH, we performed the conjugation using maleimide-modified fluorophores that bind directly to the C-terminal cysteine at the VHH. Our data nicely show that the injection of two tumour-specific VHHs that carry the same NIR fluorophore results in a higher T/B ratio than the injection of a single tumour-specific VHH combined with an irrelevant VHH. This resulted in a clear tumour delineation at early time points p.i. Interestingly, significant differences in T/B ratios were also obtained between the control groups, indicating that HER2-targeted VHH injections result in a higher T/B ratio than imaging with CAIX-targeted VHH. This result may be explained by the differences in expression level of the different targeted molecules. In fact, immunohistochemical analysis of HER2 expression showed that it is homogenously distributed throughout the tumours, in contrast to CAIX whose presence was limited to the peri-necrotic region [17]. This result suggests that the application of more than one tumour marker-specific VHHs could overcome intra-tumoural heterogeneity and improve T/B ratios even further. The small size of the VHHs is particularly important for this application as it allows simultaneous binding of different VHHs to neighbouring molecules, most likely without steric hindrance. For translational purposes, the number of combined VHH probes may be limited by the maximum dose possible to administer for imaging purposes, and the clinical testing may be prolonged by the need for single-probe testing prior to the cocktail testing in patients. Thus, considering the path each single imaging probe should follow before it is approved for first-in-human testing, it may be relevant to consider the investigation of bispecific VHHs, i.e. two independent VHHs fused in one molecule. Important for this will be to determine whether a bispecific VHH can distribute throughout tissues as well as the combination has shown to distribute in these studies. In addition, non-competitive VHHs targeting the same tumour marker could be combined as simultaneous binding of different VHHs to the same target molecule would likely lead to even better T/B ratios and consequently to an improved tumour detection.

The current diagnostic ‘gold standard’ in biomarkers’ expression assessment relies exclusively on ex vivo methods, such as immunohistochemistry (IHC) and gene amplification-based fluorescent in situ hybridization (FISH). To obtain tissue for these assessments, a biopsy is required. This invasive procedure may have side effects, such as bleeding, and it is difficult to take many biopsies or repeat the procedure for monitoring the treatment response [18]. Furthermore, taking biopsies from metastases to assess receptor profiles is now common but is an even greater burden to the patient as many sites are not easily accessible [19]. Due to these limitations, non-invasive alternatives would be preferred to the current protocols. In this context, we have also investigated the potential of using two probes, targeting two independent and validated tumour markers with two distinct fluorophores, to facilitate tumour characterization and observation of different areas of the tumour. This proof of principle study demonstrates that in vivo dual-spectral imaging of two independent tumour markers, namely CAIX and HER2, is feasible. An important implication of this result is that it enables the simultaneous determination of the expression status of two tumour markers, both in a non-invasive manner in vivo, as well as ex vivo. Here, the choice of NIR fluorophore to conjugate to each VHH was carefully made, based on the requirements of each VHH (e.g. site-directed conjugation) and the available fluorophores that are compatible with the imaging system employed. Importantly, the fluorescence of the selected fluorophores was detected by the independent channels, without interferences (Additional file 2). Interestingly, we have observed that the T/B ratios differed by a factor of 2 in the case of 11A4-680 and 11A4-800 (Figs. 3 and 4). This can be explained by a difference in background fluorescence levels (Additional file 1: Figure S2), which were slightly higher in the case of 11A4-800 (726 ± 138 vs 395 ± 120). The reason for this is not clear; however, it seems to be related to differences in distribution and clearance of the IRDye800CW, compared to the IRDye680RD.

To our knowledge, this is the first study in which VHHs were used in a dual-spectral imaging setting. The idea of imaging with a combination of probes has been earlier investigated by Barrett et al. with the use of mAbs targeting epidermal growth factor receptor (EGFR) and HER2 conjugated to NIR fluorescent dyes such as Rhodamine green, Cy5.5 or Cy7 [20]. The tumour delineation was possible only 24 h p.i. of the mAb combination. Koyama et al. presented a study in which optical imaging was performed with three different mAbs, namely cetuximab (anti-EGFR), trastuzumab (anti-HER2) and daclizumab (anti-IL-2Rα), conjugated to three different fluorophores. Also in their study, optimal T/B ratios were obtained only 24 h p.i. [21]. Similar results were obtained by Sano et al. [22]. Xie et al. compared four different NIR fluorescent probes in a subcutaneous xenograft mouse model 24 h p.i. to bioluminescent and IHC data concluding that optical molecular imaging is a feasible approach to enable the detection of different tumour features simultaneously [23]. In these studies, optimal imaging conditions were obtained not earlier than 24 h p.i. Importantly, our data show that VHHs offer sufficient target specificity enabling dual-spectral imaging providing optimal contrast for tumour detection already 2–4 h after injection.

Crucial for image-guided surgery is the clear delineation of the tumour and determination of the margins. As could be expected, after removal of the skin from the fourth mammary glands, higher T/B ratios were obtained as compared to T/B ratios obtained in a non-invasive imaging setting. These observations highlight the potential of dual- or even multispectral imaging in the surgical setting, allowing for complete tumour visualization in order to enable radical tumour resections. However, at wavelengths shorter than 800 nm, more autofluorescence can be expected. It is therefore essential that the development of probes suitable for dual- or even multispectral imaging is followed by improvement in imaging systems that would provide imaging with accurate separation of each signal.

Similarly to many other cancers that can evolve into metastatic disease, breast cancer is a clinically heterogeneous disease, which develops distant metastasis in 10–15 % of breast cancer patients during 3 years postdetection of the primary tumour [24]. Based on autopsy data, the most common sites for metastatic spread are the bone, lung and liver [24–26]. With the proof of principle dual-spectral analysis of the MCF10DCIS lung metastasis model, we could detect small metastases in the millimetre range. We expect that the simultaneous targeting of several different tumour markers might further improve the detection of small metastases, which could have a great importance in the surgical setting. For whole-body imaging and initial detection of metastases, VHHs could possibly be employed with nuclear imaging, as suggested recently by Keyaerts and co-workers [16]. Metastases may differ in the expression of tumour markers compared to the primary tumour [19]; therefore, this approach could increase the probability of detecting the metastases in a surgical setting and in addition provide the information of which tumour markers are predominant. In such a setting, a general ‘cocktail’ of different tumour markers—the most frequently present markers in all breast cancers—could be sufficient to detect most, if not all, cancerous lesions. The use of different fluorophores in such a ‘cocktail’ could provide further information on the molecular heterogeneity of the tumours. In relation to this, we recently performed a study to determine which tumour markers would be sufficient to image most of breast cancers. The combination of HER2, CAIX, GLUT1, EGFR, IGF1-R and MET could detect 45.5 % of tumours, including basal/triple negative and HER2-driven ductal cancer [10]. The addition of markers with a twofold T/B ratio could increase the detection rate to 98 % [10].

Furthermore, multispectral optical imaging is a step forward towards implementation in the clinic of the recently introduced concept of molecular fluorescence pathology, as a cost-effective, time-efficient and sensitive technique, complementary to conventional IHC. Direct fluorescence imaging of the VHHs conjugated to NIR fluorophores already present in the tumour tissue would require no further costly lab processing, but only sectioning of the tissue blocks and subsequent imaging. This molecular fluorescent pathology would, however, strongly depend on the imaging target and probably be restricted to cell surface markers that are accessible to VHHs.

## Conclusions

With this feasibility study, we show the successful optical molecular imaging of CAIX- and HER2-positive DCIS xenografts employing a combination of two tumour-specific VHHs, in vivo, in the mimicked surgical setting, as well as ex vivo on tumour sections. The simultaneous determination of the expression status of multiple, clinically relevant tumour markers could lead to a better detection of the primary tumour and its metastases, providing optimal tumour delineation during surgery under image guidance. Furthermore, this approach could provide a more rapid assessment of tumour marker expression in the pathological setting. Altogether, although this is a proof of principle study and further studies are certainly needed to assess the potential of this approach in the clinic, multispectral optical imaging could possibly improve the current management of patients from early diagnosis of cancer to surgical procedures, treatment decision-making and monitoring of treatment response.

## Additional files

Additional file 1:
**Supplementary methods and figures.** Figure 1S: SDS-PAGE analysis of NIR fluorescent VHHs. Figure 2S: Total fluorescence intensity of 11A4-800 and 11A4-680 at the tumour and in the background area obtained 5 h p.i. during the ex vivo tumour imaging. (34.9 MB) Additional file 2:
**Signal bleeding.** Each fluorescent VHH is detected by one channel only of the imaging system. (4.16 MB)

## Abbreviations

CAIX: carbonic anhydrase IX
CT: computed tomography
DCIS: ductal carcinoma in situ
DMEM/F12: Dulbecco’s modified Eagle medium nutrient mixture F-12
EGFR: epidermal growth factor receptor
ESM: electronic supplementary material
FCS: fetal calf serum
FISH: fluorescent in situ hybridization
GLUT1: glucose transporter 1
H&E: haematoxylin and eosin
HER2: human epidermal growth factor receptor 2
IGF1-R: insulin-like growth factor 1 receptor
IHC: immunohistochemistry
mAbs: monoclonal antibodies
MRI: magnetic resonance imaging
NIR: near-infrared
p.i.: postinjection
PET: positron emission tomography
SDS-PAGE: sodium dodecyl sulfate polyacrylamide gel electrophoresis
SPECT: single-photon emission tomography
T/B: tumour-to-background
US: ultrasound
VHH: variable domain of the heavy chain from heavy-chain antibodies
