# Supplementary material for: Optical imaging of pre-invasive breast cancer with a combination of VHHs targeting CAIX and HER2 increases contrast and facilitates tumour characterization
Source: EJNMMI Res. 2016 Feb 10;6:14. doi: 10.1186/s13550-016-0166-y (PMC4747965; doi:10.1186/s13550-016-0166-y)
Supplement: Additional file 1: — Supplementary methods and figures. Figure 1S: SDS-PAGE analysis of NIR fluorescent VHHs. Figure 2S: Total fluorescence intensity of 11A4-800 and 11A4-680 at the tumour and in the background area obtained 5 h p.i. during the ex vivo tumour imaging. (34.9 MB) [file 13550_2016_166_MOESM1_ESM.docx]

EUROPEAN JOURNAL OF NUCLEAR MEDICINE AND MOLECULAR IMAGING RESEARCH

**Electronic Supplementary Material**

Optical imaging of breast cancer with a combination of VHHs targeting CAIX and HER2 increases contrast and facilitates tumour characterization

Marta M. Kijanka^1*^, Aram S.A. van Brussel^1,2*^, Elsken van der Wall^3^, Willem P.T.M. Mali^4^, Paul J. van Diest^2^, Paul M.P. van Bergen en Henegouwen^1^, Sabrina Oliveira^1,2^

^1^Division of Cell Biology, Department of Biology, Science Faculty, Utrecht University, Utrecht, The Netherlands

^2^Department of Pathology, University Medical Center Utrecht, Utrecht, The Netherlands

^3^Division of Internal Medicine and Dermatology, University Medical Center Utrecht, Utrecht, The Netherlands

^4^Department of Radiology, University Medical Center Utrecht, Utrecht, The Netherlands

^*^ Authors contributed equally

### Corresponding author:

### Sabrina Oliveira, PhD

Molecular Oncology group, Division of Cell Biology

Department of Biology, Science Faculty, Utrecht University

Padualaan 8, 3584 CH, Utrecht, The Netherlands

Phone: +31 30 253 5421

Email: S.Oliveira@uu.nl

**Supplementary Methods**

**Gel electrophoresis of fluorescent VHHs**

Samples of fluorescent VHHs (0.5 μg) were size separated on a 15% polyacrylamide gel. The gel was immediately imaged on the Odyssey scanner (LI-COR) using the 700 nm channel for detection of IRDye680RD and IRDye700DX, while the 800 nm channel was used to image the IRDye800C.

**Supplementary Figures**

**
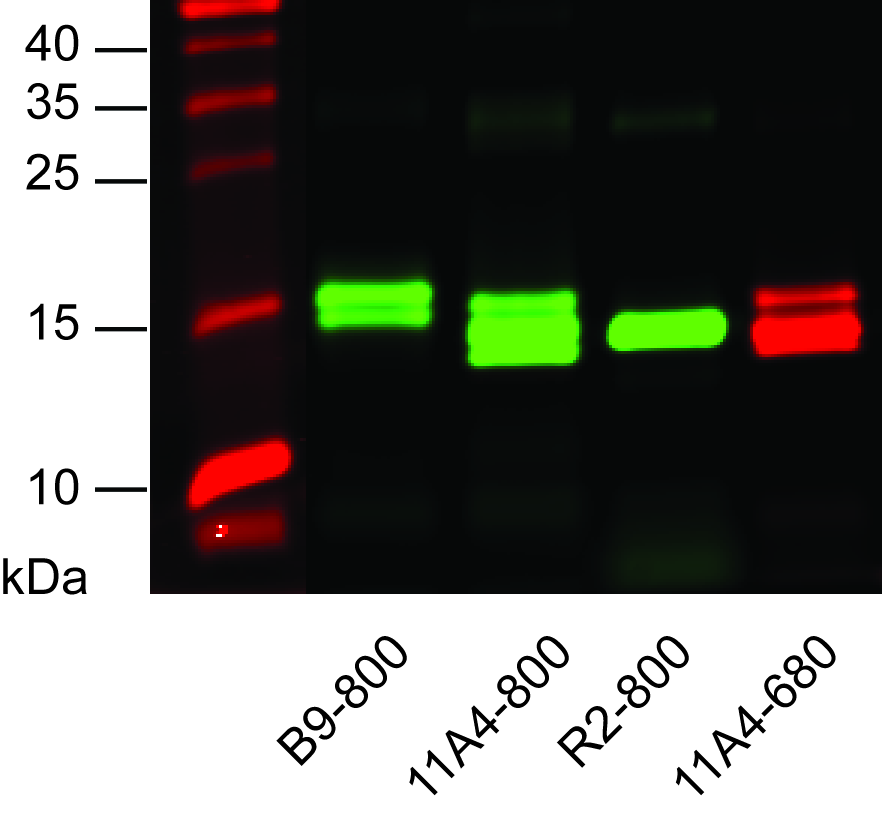
**

**Fig.1S** SDS-PAGE analysis of NIR fluorescent VHHs

**Fig.2S** Total fluorescence intensity of 11A4-800 and 11A4-680 at the tumour and in the background area obtained 5 h p.i. during the *ex vivo* tumour imaging. The difference between background fluorescence levels of 11A4-680 and 11A4-800 was detected (* p=0.0286)
